# Phenolic Composition of Artichoke Waste and Its Antioxidant Capacity on Differentiated Caco-2 Cells

**DOI:** 10.3390/nu11081723

**Published:** 2019-07-25

**Authors:** Nerea Jiménez-Moreno, María José Cimminelli, Francesca Volpe, Raul Ansó, Irene Esparza, Inés Mármol, María Jesús Rodríguez-Yoldi, Carmen Ancín-Azpilicueta

**Affiliations:** 1 Department of Sciences, InaMat, Universidad Pública de Navarra, Campus Arrosadía s/n, 31006 Pamplona, Spain; 2 Department of Pharmacology and Physiology, Veterinary Faculty, C/ Miguel Servet 177, University of Zaragoza, CIBERobn (ISCIII), IIS Aragón, IA2, 50013 Zaragoza, Spain

**Keywords:** artichoke waste, valorization, polyphenols, antioxidant capacity, Caco-2 cells

## Abstract

Artichoke waste represents a huge amount of discarded material. This study presents the by-products (bracts, exterior leaves, and stalks) of the “Blanca de Tudela” artichoke variety as a potential source of phenolic compounds with promising antioxidant properties. Artichoke residues were subjected to different extraction processes, and the antioxidant capacity and phenolic composition of the extracts were analyzed by spectrophotometric methods and high performance liquid chromatography (HPLC) analyses, respectively. The most abundant polyphenols in artichoke waste were chlorogenic acid, luteolin-7-*O*-rutinoside, and luteolin-7-*O*-glucoside. Minor quantities of cynarin, luteolin, apigenin-7-*O*-glucoside, apigenin-7-*O*-rutinoside, and naringenin-7-*O*-glucoside were also found. The antioxidant activity of the obtained extracts determined by ABTS [2, 2′-azinobis (3-ethylbenzothiazoline-6-sulphonic acid)], DPPH (2,2-diphenyl-1-pycrilhydracyl), and FRAP (Ferric Ion Reducing Antioxidant Power) was highly correlated with the total concentration of phenolic compounds. Chlorogenic acid, luteolin-7-*O*-glucoside, and luteolin-7-*O*-rutinoside, the most abundant compounds in 60% methanol extracts, are the components most responsible for the antioxidant activity of the artichoke waste extracts. The extract with the best antioxidant capacity was selected to assay its antioxidant potential on a model intestinal barrier. This action of the hydroxycinnamic acids on intestinal cells (Caco-2) was confirmed. In summary, artichoke waste may be considered a very interesting ingredient for food functionalization and for therapeutic purposes.

## 1. Introduction

The agri-food industry generates millions of tons of waste each year, which leads to serious environmental problems and a great loss of very valuable biocompounds [1]. Vegetable waste has a high content of bioactive compounds, such as antioxidants and fiber. Therefore, its use for developing functional food, food additives, or nutraceuticals would be very interesting. Furthermore, the growing demand for high-quality bio-products means that more and more attempts are being made to replace synthetic additives by natural ones. Therefore, it is very important to obtain these additives from raw materials and not to destroy foods in the process [2,3,4,5].

Artichoke (*Cynara scolymus* L., family *Asteraceae*) is a herbaceous perennial plant which originated in the Mediterranean area, but which is now widely grown all over the world. France, Spain, and Italy comprise 80% of the total worldwide production and the wide consumption of artichoke makes it an important produce for these Mediterranean countries. In artichokes, the edible parts of the plant are large immature inflorescences, named *capitula* or heads. During the industrial processing of artichoke, about 60–85% of harvested vegetal is discarded [6,7]. Artichoke waste mainly consists of the external parts of the heads, which are commonly known as bracts, as well as the leaves and stems. These residues are a source of environmental contamination and also a great economic loss as they are a very rich source of bioactive phenolic compounds, inulin, fiber, and minerals [8,9]. Artichoke has been used as both a food and medicine thanks to its beneficial effects against hepato-biliar diseases and as a digestive aid since ancient times [10]. It also delays the oxidation of low-density lipoproteins (LDL) as it helps prolong the lag phase in the formation of conjugated diene and inhibits the propagation phase [11]. These health benefits derived from the consumption of this plant are mainly thanks to its content of polyphenols [12]. Although the composition of artichoke depends on many factors (variety, climate, harvest time etc.), the main phenolic compounds present in this vegetable and, consequently in its waste, are hydroxycinnamic acids and flavonoids [13,14]. These compounds, found in abundance in artichoke waste, represent a very important potential source of nutraceutical and food additives [15]. The existence of phenolic compounds in the human diet correlates with a protective effect against certain chronic and degenerative diseases related to oxidative stress [16]. Polyphenols are able to reduce the abnormally increased levels of reactive oxygen species (ROS) which are found in a wide range of disorders, including inflammatory bowel disease and cancer [17]. The synthesis of molecules with an antioxidant ability such as phenolic compounds is very complex, so their extraction from natural sources is one of the main beneficial strategies. Therefore, the value of artichoke waste for the extraction of this type of compound could be of great interest.

Larrossa et al. [18] and Llorach et al. [19] used water phenolic extracts obtained from artichoke waste to functionalize tomato juice and commercial chicken soup, respectively. In both cases, they managed to increase the antioxidant activity of the functionalized food within consumers’ acceptance limits. Pasqualone et al. [20] enriched fresh pasta with an artichoke extract that was rich in phenolic compounds and observed that the pasta enriched with the extract showed higher phenolic compounds and antioxidant activity than the control pasta. However, much of the research has focused on the antioxidant activity of artichoke leaf extracts instead of the global artichoke processing waste, which may constitute an alternative to the commercial leaf extracts currently available for liver care and cholesterol metabolism.

In Spain, the most important artichoke variety is called “Blanca de Tudela”. This variety has a high quality and an important economic impact within the Ebro valley region. However, the industries that are currently processing this vegetable produce a high level of artichoke waste, with a corresponding negative environmental impact and economic loss. This variety of artichoke is a plant of early production and high productivity, with a rounded inflorescence, without spikes in the bracts, and with a circular hole in the upper part. The aim of this study was to determine the composition of polyphenols of waste extract from the “Blanca de Tudela” artichoke and evaluate its antioxidant capacity using a model of the intestinal mucosa (differentiated Caco-2 cells) upon oxidative stress damage induced by hydrogen peroxide insult, in order to determine its potential application for oxidative stress-related disorders of the gastrointestinal tract.

## 2. Materials and Methods

### 2.1. Raw Material and Extraction Process

The artichokes used in this work were the *Cynara cardunculus* subspecie *scolymus* “Blanca de Tudela” variety, from the spring harvest (year 2017). Artichokes were washed with tap water and manually cleaned and dried. Subsequently, the non-edible parts, such as external bracts, leaves, and stems (waste material), were separated from the edible part of the artichoke. Afterwards, artichoke waste was mixed together, cut up into small pieces, stored frozen at −20 ºC, and freeze-dried (freeze-dryer, Telstar Cryodos, Madrid, Spain). 

**Figure 1:**
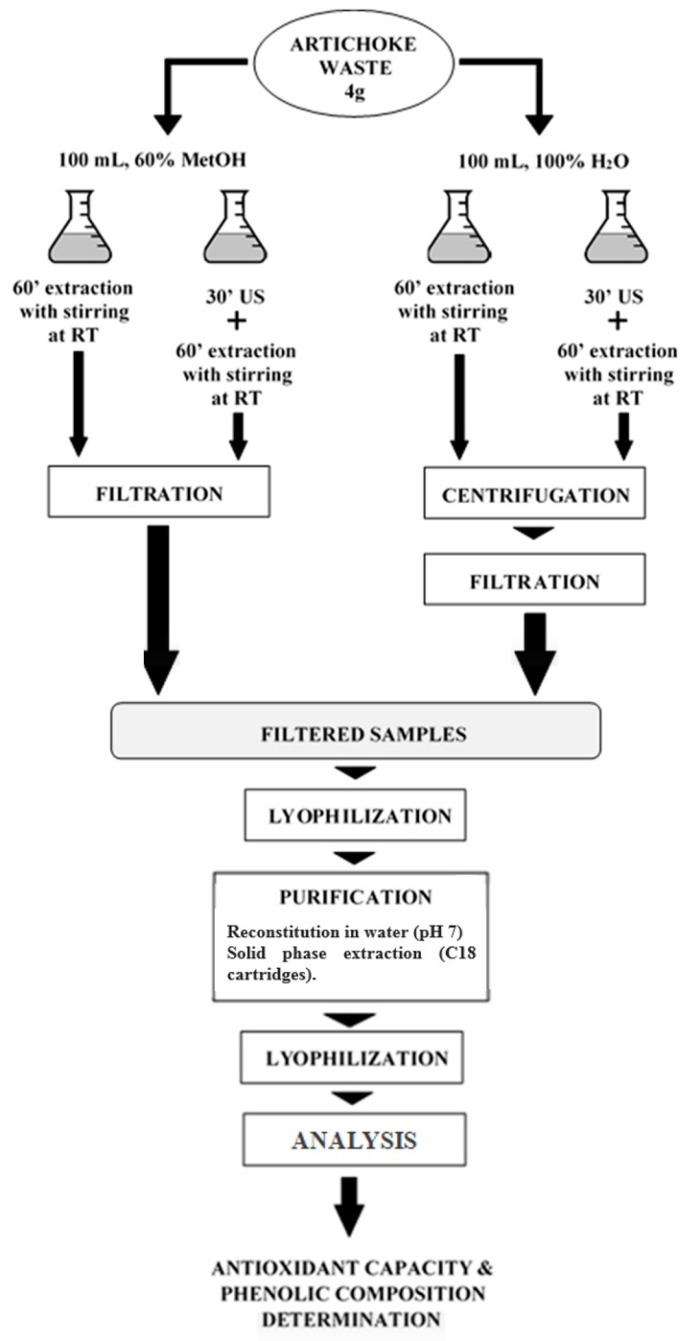
Diagram of the integral valorization of artichoke waste samples conducted to obtain antioxidant extracts.

The extraction of phenolic compounds from artichoke waste was performed using two different solvents, water and methanol:water (60:40, v/v) (Scharlab, Sentmenat, Spain), with and without ultrasound application. Figure 1 shows a diagram of the process applied to artichoke waste samples in order to obtain the extracts. To extract the phenolic compounds, 4 g of freeze-dried artichoke waste was mixed with 100 mL of solvent. In the case of the extractions without ultrasound, the sample/solvent mixture was macerated for 1 h with stirring at room temperature. When ultrasound was applied, the mixture was treated for 30 min in an ultrasound bath (Ultrasons, Selecta, Barcelona, Spain) prior to the maceration step. Subsequently, the samples extracted with 60% methanol were filtered and stored at −20 ºC. In the case of the extracts obtained with water as extraction solvent, they were centrifuged for 15 min at 8000 rpm (Sorvall ST 8, Thermo Scientific, Waltham, Massachusetts, USA) and subsequently, they were filtered and stored at −20 ºC. The liquid fraction was freeze-dried and part of the extract obtained after freeze-drying was stored at −20 ºC for a later analysis of its antioxidant capacity and total phenolic content (TPC). The rest of the extract was purified and fractioned into hydroxycinnamic acids and flavonoids, following the method described by Lombardo et al. [21]. Briefly, the extract was reconstituted in MilliQ water and was adjusted to pH 7. Afterwards, a solid phase extraction using ExtraBond C18 cartridges (Scharlab, Barcelona, Spain) was performed. The hydroxycinnamic acids were eluted using methanol at 10% (v/v), and flavonoids with 100% methanol. Both fractions were freeze-dried. Hydroxycinnamic acids (fraction 1) and flavonoids (fraction 2) were reconstituted with 4 mL and 1 mL of methanol:water 50% (v/v), respectively. All the samples were kept at −20 ºC until their later analysis. The extraction yields obtained by the different extraction procedures are shown in Table S1 (Supplementary Material).

### 2.2. Determination of Phenol Compounds of the Extracts by High Performance Liquid Chromatography

Analyses of each fraction were performed with Waters high-pressure liquid chromatography (Waters) equipped with two 510 pumps, a 717 Plus autosampler, and a Photodiode Array 996 detector programmed at different wavelengths (from 200 to 600 nm). A reverse phase column was used (Phenomenex Synergi HydroRP, 150 × 3 mm, particle size of 4 μm) at 25 °C. The instrument control and data processing were carried out with Empower 2.0 software. In Table 1, the chemical formulas, retention time, and wavelength of each of the phenolic compounds analyzed are shown. 

For the chromatographic analyses of the phenolic compounds in artichoke waste, a modified method of Lombardo et al. [21] was used. Two mobile phases, A (2% acetic acid) and B (acetonitrile and 2% acetic acid, 50:50, v/v), were used for both analyses. The flow rate was 1 mL/min and the injection volume was 10 μL. All the HPLC quality solvents were from Scharlab (Barcelona, Spain). Identification of the compounds was carried out by a double coincidence of the UV-Vis spectrum at the characteristic wavelength of each compound, and the retention time of its corresponding standard. All the standards used were from Sigma-Aldrich (Madrid, Spain). Quantification of the antioxidants was carried out using calibration curves for each compound analyzed. In all cases, the coefficient of lineal correlation was R^2^ > 0.99.

### 2.3. Antioxidant Capacity and Total Phenol Content of the Extracts Obtained from Artichoke Waste

The antioxidant capacity of the different extracts was determined by the ABTS radical scavenging assay, DPPH radical scavenging assay, and FRAP (Ferric Ion Reducing Antioxidant Power). The ABTS method [2, 2′-azinobis (3-ethylbenzothiazoline-6-sulphonic acid)] used is based on the method outlined by Re et al. [22]. The calibration curve was made from a 5 mM solution of Trolox, ranging from 0.05 to 2.4 mM. The absorbance was determined at 734 nm with a UV/Vis spectrometer (Jenway 7315, Staffordshire, UK).

The DPPH assay (2,2-diphenyl-1-pycrilhydracyl) used is based on the method outlined by Brand-Williams et al. [23]. For the calibration curve, Trolox was used in different concentrations ranging from 0.05 mM to 0.95 mM. For the antioxidant capacity determination, 150 μL of each artichoke waste extract was added to 2.85 mL of the DPPH stock solution and after 30 min in darkness, the absorbance at 515 nm was measured.

The antioxidant capacity of extracts was also determined by the FRAP assay proposed by Benzie and Strain [24]. Known concentrations of Trolox, in the range of 0.05–0.95 mM, were used for preparing the calibration curve. The absorbance of each standard and the extract was measured at 595 nm.

Finally, the total phenolic content (TPC) was analyzed using the Folin–Ciocalteu method outlined by Singleton et al. [25]. Briefly, solutions of gallic acid with different concentrations ranging from 0.2 mM to 3.2 mM were prepared, in order to obtain the calibration curve. The absorbance was measured at 765 nm. In all cases, the extracts were analyzed in triplicate.

### 2.4. Biological Assays

#### 2.4.1. Cell Culture

The human colorectal adenocarcinoma Caco-2 cell line (TC7 clone) was kindly provided by Dr. Edith Brot-Laroche (Université Pierre et Marie Curie-Paris 6, UMR S 872, Les Cordeliers, France). Cells were maintained in a humidified atmosphere of 5% CO_2_ at 37 ºC. Cells (passages 20–50) were grown in Dulbecco’s Modified Eagles medium (DMEM) (Gibco Invitrogen, Paisley, UK) supplemented with 20% fetal bovine serum, 1% non-essential amino acids, 1% penicillin (1000 U/mL), 1% streptomycin (1000 μg/mL), and 1% amphotericin (250 μg/mL). Cell medium was replaced every three days and cells were passaged once a week with 0.25% trypsin-1 mM EDTA. Cell confluence (80%)—differentiated cells—was confirmed by microscopic observance. 

#### 2.4.2. Measurement of Cell Proliferation

Cells were seeded on 96-wells plates at a density of 4000 cells per well and they were incubated under standard culture conditions until differentiation (7 days). Cell confluence (80%) was confirmed by microscopic observance. For treatment, lyophilized fractions 1 and 2 from extracts obtained with methanol:water 60% (v/v) and ultrasounds were weighed and dissolved in cell culture medium (without fetal bovine serum) to the required concentrations: 1000, 500, 250, 125, and 62.5 μg/mL. These concentrations were chosen in relation to previous studies in our laboratory [2,17]. Cells were incubated with the extracts for 72 h and changes in cell proliferation were determined by the sulforhodamine B assay, as described by Jiménez et al. [17]. IC_50_ was defined as the concentration of extract that reduced cell viability to 50%.

#### 2.4.3. Measurement of Intracellular Reactive Oxygen Species Levels

Cells were seeded on 96-well plates at a density of 4000 cells per well and were incubated under standard culture conditions until differentiation (7 days). For treatment, extracts were dissolved on cell culture medium (without fetal bovine serum) to the required concentrations: 1000, 500, and 250 μg/mL. Cells were incubated with the extracts for 1 or 2 h. Then, cells were incubated for 20 min with 20 mM 2′,7’-dichlorofluorescein diacetate (DCFH-DA) at 37 °C protected from light, according to the assay previously described by Sánchez-de-Diego et al. [26]. For hydrogen peroxide insult, the cell culture was then replaced and 20 mM H_2_O_2_ (dissolved on PBS: Phosphate Buffer Solution) was added to each well except negative control cells, which were incubated with cell culture medium. After 20 minutes incubation with H_2_O_2_ protected from light, the fluorescence intensity was measured using a FLUOstar Omega Microplate Reader (BMG Labtech, Ortenberg, Germany). Excitation and emission wavelength settings were 485 and 535 nm, respectively. The intensity of fluorescence is considered as a reflection of the total intracellular ROS levels.

### 2.5. Statistical Analysis

Data are presented as the mean ± SE. Data were subjected to a two-tailed t-test and differences were considered significant at p ≤ 0.05. Statistical analysis was carried out using IBM SPSS software v 23 (New York, NY, USA). 

## 3. Results and Discussion

### 3.1. Phenolic Composition of the Extracts Obtained from Artichoke Waste

Table 2 shows the phenolic composition of the extracts obtained from the different extraction methods applied. The phenolic profile was quite different, both qualitatively and quantitatively, depending on the extraction method used. The richest extract in phenolic compounds was obtained using methanol:water (60% v/v) with ultrasound application. Methanol was much more efficient than water for extracting both hydroxycinnamic acids and flavonoids. Among the hydroxycinnamic acids, caffeic acid was not detected in any sample and chlorogenic acid (5-O-caffeoylquinic acid) was the predominant compound in all of them. The absence of caffeic acid in all the extracts agrees with previous findings by Schütz et al. [27], who explained the formation of this compound from the hydrolysis of mono- and di-caffeoylquinic acids during processing. Taking into consideration that the caffeic acid inhibits the proliferation of fibrosarcoma [28] and of breast cancer cells [29], and that it acts favorably on the metabolism of glucose, suppressing hepatic gluconeogenesis and hyperglycemia [30], it would be interesting to perform further studies in order to more precisely determine what conditions favor this hydrolysis. Caffeoylquinic acids are the most abundant hydroxycinnamic acids in artichoke, especially chlorogenic acid [31]. During the storage of artichoke heads at temperatures below 4 °C, there is a biosynthetic increase in phenolic compounds, especially of chlorogenic acid, since the activity of the enzyme phenylalanine-ammonia lyase is induced. The chlorogenic acid produces a colorless complex with Fe^2+^, but the oxidant conditions of the medium induce the formation of a complex of chlorogenic acid/Fe^3+^ gray-blue, responsible for the artichoke browning [11]. Cynarin (1,3-O-dicaffeoylquinic acid) was found in a very low concentration in the samples extracted with 60% methanol, and was not detected in the samples extracted with water. This compound is the most well-known caffeoylquinic acid in artichoke extracts because it acts by stimulating biliary secretion and cholesterol metabolism [11,32]. 

The most abundant flavonoids in the extracts obtained from artichoke waste using 60% methanol were luteolin-7-*O*-rutinoside and luteolin-7-O-glucoside, while very small amounts of the rest of the flavonoids were found (Table 2). These results coincide with those found by Pandino et al. [9] in extracts obtained with 70% methanol from leaves and stems of different artichoke varieties. In the extracts obtained using water as a solvent, the flavonoids that were most extracted were luteolin-7-*O*-rutinoside and apigenin-7-*O*-rutinoside, although the extraction efficiency was much lower than when methanol was used. Luteolin derivatives are much more abundant in artichoke waste than in the edible part of this vegetable, where the most important flavonoids would seem to be the apigenin derivatives, especially apigenin-7-*O*-glucuronide [9,21,33]. Narirutin (naringenin-7-*O*-rutinoside) was not detected in the extracts obtained using 60% methanol as the extraction solvent, although when water was used in the extraction, apigenin-7-*O*-glucoside was the flavonoid that was not detected in the extracts. It is clear that the solvent type significantly affected phenolic compounds recovery. Differences in the extraction efficiency are mainly due to the higher range of polarity of 60% methanol as a solvent compared with 100% water, but other factors, such as weakening of the solute–matrix interactions and swelling of the plant material, could also be involved [34]. Narirutin and naringenin-7-*O*-glucoside provide beneficial effects for health due to their anti-cancerous and antioxidant properties [35]. The therapeutic properties of artichoke are not due to isolated compounds, but rather to several active compounds that act synergistically, and many of them are found in important concentrations in artichoke by-products. Among those compounds with synergistic action are caffeoylquinic acids and flavonoids, such as luteolin and luteolin 7-*O*-glucoside [36,37]. Hydroxycinnamic acids predominate in artichoke stems (68% of TPC) and flavonoids in leaves and external bracts (95 and 84% of TPC, respectively) [31], but it has been observed that the beneficial properties for health arise from the synergistic effect between both types of phenolic compounds [36,38] and hence the interest in their joint extraction to achieve an integral valorization.

The ultrasound application only increased the content of phenolic compounds when 60% methanol was used as the solvent. In contrast, Punzi et al. [39] found that the application of direct sonication for 60 minutes on samples of artichoke by-products, using water as the solvent, improved the recovery of phenolic compounds compared with untreated samples. Ultrasounds normally improve the extraction of bioactive compounds, as the cavitational effect facilitates the release of extractable compounds and enhances the mass transport by diffusion or by disrupting the plant cell walls [40,41,42]. Furthermore, mild treatment of ultrasounds does not produce any significant changes in the properties and functionality of most of the bioactive compounds [43], which makes it ideal for the extraction of antioxidants. In this study, indirect sonication was applied, in which the ultrasonic energy is transmitted through the water and into a vessel or sample tube. This is likely to be the main reason why the extraction improved very little with the ultrasound treatment, since in direct sonication, the energy is transmitted from the probe directly into the sample with a high intensity.

As far as artichoke phenolics are concerned, much of the research has focused on the antioxidant activity of artichoke leaf extracts, but in this research work, it has been shown that artichoke processing waste may constitute an alternative to the commercial leaf extracts currently available for liver care and cholesterol metabolism.

### 3.2. Antioxidant Capacity and Total Phenol Content of the Extracts Obtained from Artichoke Waste

**Figure 2:**
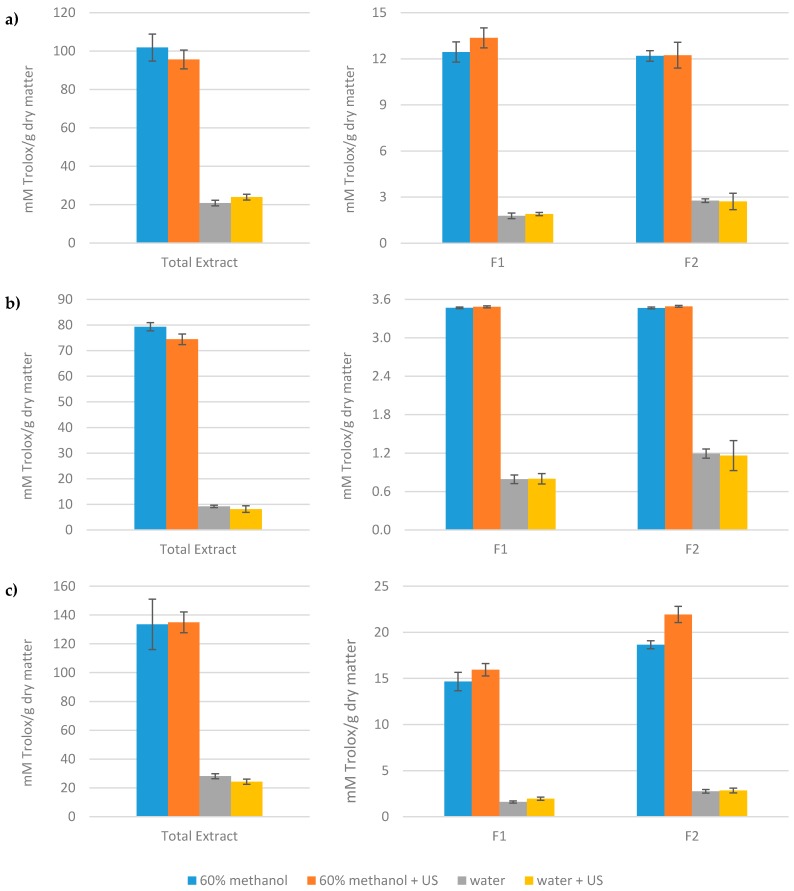
Antioxidant capacity of the extracts measured by 2, 2′-azinobis (3-ethylbenzothiazoline-6-sulphonic acid) (ABTS) (**a**), 2,2-diphenyl-1-pycrilhydracyl (DPPH) (**b**), and Ferric Ion Reducing Antioxidant Power (FRAP) (**c**) assays.

The antioxidant capacity of the different extracts obtained from artichoke waste was evaluated by the ABTS, DPPH, and FRAP methods. It is important to properly assay the antioxidant capacity of food-derived extracts because it is due to a combination of the activities of several antioxidant compounds. Figure 2 shows the results of antioxidant capacity obtained for all the extracts by means of these methods. For all the methods assayed, the antioxidant capacity of the extracts obtained using 60% methanol as the extraction solvent was much higher than those extracts obtained with water. On the other hand, the antioxidant capacity of the extracts obtained from ultrasound-assisted extraction was very similar to the antioxidant capacity of the samples obtained by single solvent extraction. These results coincide with the results on the content of phenolic compounds in the different artichoke waste extracts (Table 2). In fact, the results of total phenolics calculated as the sum of the concentration of all the identified compounds in the chromatographic analyses are highly and significantly correlated with the values of antioxidant capacity found with the three different assays of antioxidant capacity (Pearson correlation R of 0.972, 0.974, and 0.985 for ABTS, DPPH, and FRAP assays, respectively). These results seem to indicate that chlorogenic acid, luteolin-7-*O*-glucoside, and luteolin-7-*O*-rutinoside, the most abundant compounds in 60% methanol extracts, are the components most responsible for the antioxidant activity of the artichoke waste extracts. Additionally, Fritsche et al. [44] found that luteolin, luteolin derivatives, and chlorogenic acid showed the strongest antioxidant effect among the different components of artichoke leaf extracts. Caffeoylquinic acids exhibit important antioxidant activity, as has been demonstrated by different authors [31,32,45,46]. Likewise, the copper chelating properties of luteolin and its derivatives suggest that they have a very important role in the antioxidant effects of artichoke [11]. Consequently, the antioxidant activity of artichoke extracts must be due, in part, to their content in flavonoids, which act as hydrogen donors and are metal ion chelators [47]. 

The antioxidant capacity of the total extract was much greater than the sum of the antioxidant capacities of fractions 1 (hydroxyxinnamic acids) and 2 (flavonoids) in all cases. This seems to indicate that there exists an important synergistic and/or additive action between the antioxidant compounds and other compounds present in the extract [48,49]. The antioxidant activity of fractions 1 and 2 was very similar when using ABTS and DPPH assays; however, when using the FRAP assay, the flavonoid fraction (fraction 2) had higher values of antioxidant capacity than the fraction rich in hydroxycinnamic acids (fraction 1). Moreover, with this method, the extracts of fraction 2 obtained by 60% methanol ultrasound-assisted extraction showed a greater antioxidant capacity than those obtained with the same solvent, but without ultrasound treatment. This result coincides with the greater content of luteolin derivatives found by means of HPLC-DAD analyses in the extracts obtained using 60% methanol and ultrasound-assisted extractions (Table 2). Moreover, among the antioxidant capacity methods used in this work, the FRAP assay is the only method with a high and significant (*p* < 0.05) correlation with the content of luteolin-7-*O*-rutinoside (Pearson R of 0.962), the most abundant flavonoid in our artichoke waste extracts. 

The values of antioxidant capacity obtained with ABTS and FRAP assays were found within the same range, both for the total extract, as well as for the fractions rich in hydroxycinnamic acids and flavonoids. On the other hand, the antioxidant capacity obtained by means of the DPPH assay was lower in all cases. DPPH and ABTS methods are normally considered electron transfer-based assays, but hydrogen atom transfer also takes place in both methods, although in the DPPH assay, this mechanism is more marginal [50]. The FRAP assay shows a different mechanism of action since there are no free radicals implicated in the reaction, but rather it is based on the ability of the antioxidants to reduce ferric iron (Fe^3+^) to ferrous iron (Fe^2+^). The redox potential of the ferric tripyridyltriazine complex (0.70 V), which is involved in the FRAP reaction, is comparable to that of the ABTS^●+^ radical (0.68 V), and consequently, similar compounds will react in both ABTS and FRAP assays [51]. Furthermore, although the DPPH assay is a simple and rapid method for antioxidant screening, interpretation is complex, as it shows some drawbacks. On the one hand, there are compounds with spectra that overlap DPPH at 515 nm [50,52], and on the other hand, the steric accessibility is also a major limiting element in the reaction [51,53]. This is because the radical site is protected inside a reaction cage formed by the two phenyl rings orthogonal to each other, and the picryl ring angled at about 30° with its two nitro groups oriented above and below the radical site [54]. The low values of the antioxidant capacity observed when using the DPPH assay could be due to both factors. In fact, Xie and Schaich [54] did not recommend the DPPH assay to evaluate the potential antioxidant activity of plant extracts where compounds with different structures are present.

**Figure 3:**
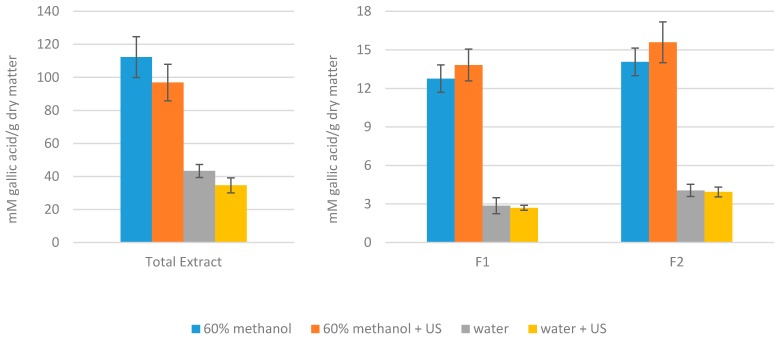
Total phenolic content (TPC) of the extracts determined by the Folin–Ciocalteu method.

Folin–Ciocalteu reagent has been used for a long time to measure the total content of phenolic compounds in different samples, in spite of the fact that some non-phenolic compounds can also reduce this reactive, thus overestimating the TPC of the sample [55]. Figure 3 shows the results of TPC expressed as mM gallic acid equivalents per g of dry matter. The results obtained are well-correlated to those obtained in the antioxidant capacity assays (Figure 2). The Pearson correlation R values between the TPC obtained by Folin–Ciocalteu reagent and antioxidant capacity by ABTS, DPPH, and FRAP are very high (0.98, 0.962, and 0.97, respectively) and statistically significant in all cases (*p* < 0.01). This high correlation is not surprising, since the basic mechanism of all these methods is an oxidation/reduction reaction. Therefore, different authors recommend the use of TPC by the Folin–Ciocalteu assay to measure the antioxidant capacity of extracts as this is a simple method, which is reproducible, robust, and has a lot of comparable data in the literature [51,55].

### 3.3. Antioxidant Capacity on a Model Intestinal Barrier

Considering that the highest antioxidant capacity and polyphenol content were found for the extracts obtained with methanol:water 60% v/v and ultrasounds, both fraction 1 and 2 were selected for the analysis of their antioxidant capacity on a model of the intestinal barrier. However, due to the insolubility of fraction 2 on cell culture medium, we were unable to evaluate its antioxidant capacity toward hydrogen peroxide insult on Caco-2 cells. Given the promising results obtained with FRAP, DPPH, and ABTS assays, fraction 2 could be a strong candidate for in vivo tests with animal models and further research will be carried out in this regard. Consequently, we analyzed the potential effect of fraction 1 on the protection of the intestinal barrier upon exogenous oxidative stress.

The antioxidant capacity of fraction 1 (hydroxyxinnamic acids) was evaluated on Caco-2 cells. This cell line spontaneously acquires the phenotypic features of non-cancerous enterocytes after reaching confluence. Monolayer Caco-2 cells form tight junctions and present the cylindrical polarized morphology of enterocytes, expressing functional microvilli on the apical side [56,57,58]. Therefore, differentiated Caco-2 cells have been established as an acceptable in vitro intestinal barrier model. 

**Figure 4:**
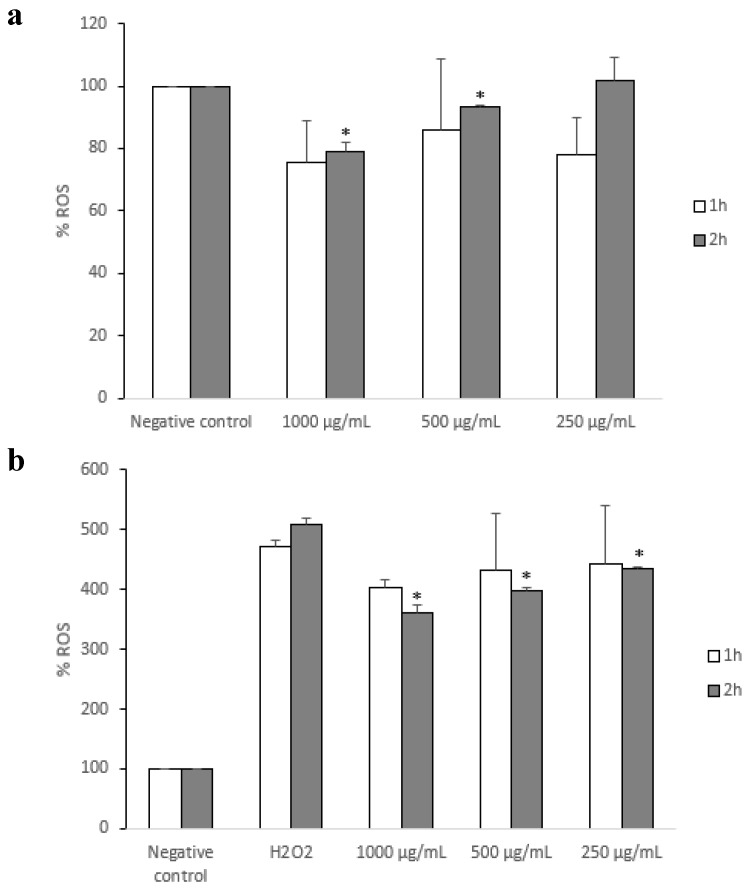
Analysis of the antioxidant capacity of fraction 1 on differentiated Caco-2 cells. (**a**) Measurement of reactive oxygen species levels after 1 and 2 h incubation with fraction 1 (1000, 500, and 250 μg/mL). (**b**) Measurement of reactive oxygen species levels after 1 and 2 h incubation with fraction 1 (1000, 500, and 250 μg/mL) and further hydrogen peroxide insult (20 min incubation with 20 mM H_2_O_2_). *p < 0.05 versus H_2_O_2_ -treated cells.

Firstly, the IC_50_ value of fraction 1 was calculated on Caco-2 cells in order to select a non-cytotoxic range of concentration. We obtained an IC_50_ value of 1023±159 μg/mL after long-term incubation (72 h). Therefore, we tested the antioxidant capacity of three concentrations below the obtained IC_50_ value (1000, 500, and 250 μg/mL) on the cells after 1 and 2 h of incubation, since such values did not display cytotoxic effects for short periods (data not shown). As can be observed in Figure 4a, we found a time- and concentration-dependent antioxidant effect of fraction 1. Although no significant changes in ROS levels were observed after 1 h for either of the evaluated concentrations, 2 h incubation with 1000 and 500 μg/mL of fraction 1 resulted in a significant decrease in intracellular peroxide production (*p* < 0.05). This data encouraged us to determine the capacity of fraction 1 to reverse hydrogen peroxide insult on differentiated Caco-2 cells (Figure 4b). Similarly, time and concentration turned out to be key factors for the antioxidant activity of fraction 1. After 2 h incubation, all tested concentrations (1000, 500, and 250 μg/mL) were able to significantly decrease H_2_O_2_-induced ROS production (*p* < 0.05), whereas such behavior was not observed after 1 h incubation. Furthermore, the highest concentration of fraction 1 tested (1000 μg/mL) displayed a greater decrease in ROS levels when compared to the lowest one (250 μg/mL), which suggests the key role of extract concentration on its antioxidant capacity. 

The antioxidant capacity of plant extracts is strongly correlated with their clinical application for the management of oxidative stress-related gastrointestinal disorders. Koláček et al. [59] found that the intake of commercially available pine bark extract Pycnogenol^®^ reduced oxidative stress biomarkers on pediatric patients of Crohn’s disease. Similarly, research performed on animal models of ulcerative colitis has revealed that the protective effect of plant extracts might be mediated by their antioxidant potential [60,61]. Our results with regard to the time- and concentration-dependent antioxidant capacity of the hydroxycinnamic acids fraction of artichoke waste extracts suggest that they might have a potential application in the management of oxidative stress-related gastrointestinal malignancies. 

Hydrogen peroxide insult is a widespread method used to mimic the pro-oxidative environment that characterizes degenerative diseases such as cancer or neurodegenerative disorders on 2D cell cultures. In this line, plant-derived extracts have been investigated for their capacity to overcome the aberrant increase in ROS levels derived from H_2_O_2_ exogenous addition. Ashwagandha, a traditional Indian herb, leaf extracts protected human neuroblastoma IMR32 cells from 2 h hydrogen peroxide insult after 24 h incubation with plant extracts at a concentration of 0.4 μg/mL [62]. Incubation of HaCaT skin cells with extracts of *Calendula officinalis* flowers prevented H_2_O_2_-induced damage, which might be interesting for the prevention of skin-related disorders, including melanoma [63]. Anthocyanins from the elderberry *Sambucus nigra* displayed a significant protective effect against various oxidative stresses on bovine aortic endothelial cells, and therefore might have a role in the prevention of cardiovascular disease [64]. Since, herein, we have demonstrated the capacity of artichoke waste extracts to overcome hydrogen peroxide insult, the therapeutic applications of these extracts might be wider and further assays are needed to evaluate their potential.

## 4. Conclusions

Chlorogenic acid, luteolin-7-*O*-rutinoside, and luteolin-7-*O*-glucoside were the antioxidants present in a greater concentration in the mixture of by-products (external bracts, leaves, and stems) from the “Blanca de Tudela” artichoke variety. Low amounts of cynarin, luteolin, apigenin-7-*O*-glucoside, apigenin-7-*O*-rutinoside, and naringenin-7-*O*-glucoside were also found. The extraction of hydroxycinnamic acids and flavonoids was much higher when 60% methanol was used as the extraction solvent in comparison to when water was used. In all cases, there was a good correlation between the concentration of total phenolics and the antioxidant capacity obtained by ABTS, DPPH, and FRAP. Of the three methods, FRAP is the only one that shows a good correlation with the concentration of the most abundant flavonoids in these extracts (luteolin derivatives), which would indicate that it is the best method for these kinds of samples. Our results also indicated that hydroxycinnamic acids from artichoke waste extracts might have a promising future in the management of oxidative stress on the gastrointestinal tract. 

## Figures and Tables

**Table 1:** Phenolic compounds analyzed: chemical structure, retention time, and detection wavelength.

	Name	Chemical Structure	Retention Time (min)	Detection Wavelength (λ, nm)
**Hydroxycinnamic Acids**	Caffeic acid	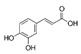	17.8	320
	Chlorogenic acid	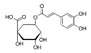	15.4	320
	Cynarin	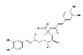	29.7	320
**Flavonoids**	Luteolin	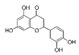	86.9	350
	Luteolin-7-*O*-glucoside	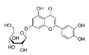	59.6	350
	Luteolin-7-*O*-rutinoside	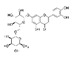	59.1	350
	Apigenin	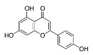	88.8	330
	Apigenin-7-*O*-glucoside	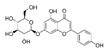	73.6	330
	Apigenin-7-*O*-rutinoside	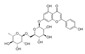	70.9	330
	Naringenin-7-*O*-glucoside	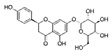	64.5	280
	Narirutin	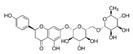	62.3	280

**Table 2:** Phenolic composition of the extracts obtained from the different extraction methods applied to the artichoke waste (μg/g dry matter of artichoke waste).

	60% Methanol (60′ Extraction)	60% Methanol (60′ Extraction + 30′ Ultrasound)	100% Water (60′ Extraction)	100% Water (60′ Extraction + 30′ Ultrasound)
**Hydroxycinnamic Acids**				
Caffeic acid	nd	nd	nd	nd
Chlorogenic acid	815 ± 50	1006 ± 113	10 ± 1	8 ± 1
Cynarin	9.8 ± 0.7	12 ± 2	nd	nd
Total hycroxycynamic acids	825	1018	10	8
**Flavonoids**				
Luteolin	5.2 ± 0.3	4.5 ± 0.9	2.4 ± 0.4	2.4 ± 0.3
Luteolin-7-*O*-glucoside	442 ± 14	469 ± 6	2.7 ± 0.1	2.9 ± 0.6
Luteolin-7-*O*-rutinoside	684 ± 66	1034 ± 20	17 ± 2	10 ± 2
Apigenin	2.46 ± 0.01	2.49 ± 0.01	3.3 ± 0.4	4.1 ± 0.6
Apigenin-7-*O*-glucoside	7.3 ± 0.1	7.2 ± 0.3	nd	nd
Apigenin-7-*O*-rutinoside	20.9 ± 0.6	20.2 ± 0.9	5.5 ± 0.3	4.6 ± 0.3
Naringenin-7-*O*-glucoside	2.96 ± 0.02	2.9 ± 0.2	2.2 ± 0.03	2.19 ± 0.05
Narirutin	nd	nd	2.14 ± 0.02	2.11 ± 0.02
Total flavonoid content	1165	1540	35	28
Total phenolic content	1990	2558	45	36

## Supplementary Materials

The following are available online at https://www.mdpi.com/2072-6643/11/8/1723/s1: Table S1: Extraction yields obtained by the different extraction procedures.
