# Supplementary material for: Phenolic Composition of Artichoke Waste and Its Antioxidant Capacity on Differentiated Caco-2 Cells
Source: Nutrients. 2019 Jul 25;11(8):1723. doi: 10.3390/nu11081723 (PMC6723629; doi:10.3390/nu11081723)
Supplement: Supplementary file 1 [file nutrients-11-01723-s001.pdf]

Table S1. Extraction yields obtained by the different extraction procedures

	μg/g dry artichoke waste		
	**Total extract**	**Fraction 1** *Hydroxycinnamic acids*	**Fraction 2** *Flavonoids*
**60% Methanol**	286 ± 6	31 ± 2	15.6 ± 0.1
**60% Methanol + US**	273 ± 20	31.2 ± 0.6	13 ± 3
**H_2O**	267 ± 15	21 ± 4	6 ± 1
**H_2O + US**	234 ± 30	21.9 ± 0.3	3 ± 1
